# Short report: Experiences of Caregivers Participating in a Telehealth Evaluation of Development for Infants (TEDI)

**DOI:** 10.1007/s10803-022-05607-x

**Published:** 2022-08-09

**Authors:** Meagan R. Talbott, Ellisa Lang, Felipe Avila, Sarah Dufek, Gregory Young

**Affiliations:** 1 MIND Institute, University of California Davis, 2825 50th Street, 95817 Sacramento, CA United States; 2 Department of Psychiatry and Behavioral Science, University of California Davis, Sacramento, USA

**Keywords:** Telehealth, Autism, Infant, Screening, Asd, Prodromal autism, Parent satisfaction, Telehealth assessment

## Abstract

A growing literature supports the feasibility and validity of telehealth-based assessments for autism spectrum disorder (ASD). Better understanding families’ experiences is crucial for sustained use beyond the COVID-19 pandemic. This study qualitatively examines caregiver experiences with the Telehealth Evaluation of Development for Infants (TEDI) protocol to better understand benefits and challenges of telehealth-based evaluations. Caregivers (*N =* 32) completed an online survey following a telehealth-based evaluation with their 6–12 month-old infants. Open-ended text responses to queries about perceived benefits, challenges, and suggestions for future adaptations were coded. Most caregivers reported positive experiences with minor feedback relating to tailoring of individual needs. Responses suggest the TEDI is a feasible approach and provide guidance for components of successful telehealth evaluations more broadly.

Many families face significant barriers accessing early evaluations for autism spectrum disorder (ASD) including a lack of timely referrals from primary care providers, long waitlists, and geographic distance from appropriately trained specialists. Families from traditionally underserved communities, including those of lower socioeconomic status and marginalized backgrounds, experience further delays in access to evaluations and services (Magaña et al., 2012; Stahmer et al., 2019; Wallis et al., 2021; Zuckerman et al., 2015). Reducing these barriers and increasing families’ access to early diagnostic and intervention services will likely require a variety of approaches to address the full range of individual families’ needs and circumstances. Telehealth approaches, in which families and clinicians connect using audio, video, and other electronic means, provide one option for increasing access to early services. Qualitative studies of early intervention practices in rural communities suggest telehealth-based approaches may be particularly useful in addressing barriers related to geographical distance, family care and work schedules, and accommodating complex medical needs (Buchter & Riggleman, 2018; Elpers et al., 2016).

A growing literature supports the cost-effectiveness and potential benefits of incorporating telehealth in diagnostic and behavioral intervention services for ASD (Juárez et al., 2018a; Knutsen et al., 2016; Stainbrook et al., 2019). Multiple studies have demonstrated the overall acceptability and efficacy of parent coaching services delivered in-person versus via telehealth (Little et al., 2018; Pickard et al., 2016; Vismara et al., 2018; Wallisch et al., 2019). The ongoing COVID-19 pandemic has further accelerated the shift to telehealth, particularly for diagnostic evaluations (Berger et al., 2021; Dahiya et al., 2021). As COVID-19 has necessitated the near ubiquitous use of telehealth to conduct diagnostic and/or eligibility assessments and to deliver early intervention services, this ‘natural experiment’ has highlighted the many ways in which telehealth may be leveraged to deliver services (Edelman, 2020; Little & Stoffel, 2021). Despite these benefits, there remain some aspects of face-to-face delivery that offer several advantages over telehealth, including administration of standardized developmental and/or cognitive measures, firsthand interaction and observation of more subtle behaviors, and experiential preference by families and/or clinicians (Corona et al., 2020; Juárez et al., 2018b; Wagner et al., 2021). Finally, the existence of a digital divide is well established, in that populations historically underserved by existing healthcare systems experience the most significant barriers accessing telehealth-delivered services (Litchfield et al., 2021; Ramsetty & Adams, 2020; Van Winkle et al., 2017). Given these limitations, it is unlikely that telehealth will – or should – fully replace face-to-face services. Instead, we should consider when, how, and for whom, telehealth may play an ongoing role in early identification and delivery of early intervention services in ASD. Answers to these questions will necessarily be guided by relevant laws, insurance and funding policies, and provider infrastructure. They should also be guided by family needs and preferences. Implementation science approaches, which involve researchers and stakeholders collaborating to develop practices that are both desirable and feasible, can help to facilitate the development and implementation of evidence-based practices in community settings (Stahmer et al., 2017). While there is a growing literature on the efficacy, feasibility, and general acceptability of telehealth in service delivery for individuals on the autism spectrum and their families, fewer studies have documented the experiences and perspectives of families using these services. Better understanding the barriers and facilitators of telehealth-based services for families is a first step in the development and implementation of programs most likely to meet their needs.

In the current paper, we sought to gain a greater understanding of telehealth-based evaluations of infants’ development by qualitatively analyzing written feedback from caregivers participating in ongoing study of one such telehealth protocol, the Telehealth Evaluation of Development for Infants (TEDI; Talbott, Dufek, Young, and Rogers 2021, Talbott et al., 2020). The TEDI protocol was initially developed prior to the COVID-19 pandemic in an effort to translate laboratory-based measures of early ASD characteristics, communication, language, play, and other related developmental domains into a telehealth format. The TEDI protocol provides detailed guidance around a set of measures and activities that can be used to conduct a social-communication-focused developmental evaluation for infants via telehealth. It involves a combination of caregiver questionnaires and a synchronous telehealth session wherein caregivers are coached through a series of play activities (e.g. free play, bubbles, peekaboo, book reading, etc.) from which primary examiner-rated and behavioral coding measures are scored. Primary behavioral measures derived from the synchronous telehealth sessions include the Autism Observation Scale for Infants (AOSI; Bryson et al., 2008), the P-ESDM Infant-Toddler Curriculum Checklist (IT-CC; Rogers et al., 2020), and the Early Communication Indicator (ECI; Greenwood et al., 2006; Greenwood et al., 2010). These measures evaluate infants’ skills in social communication, play, imitation, joint attention, play, cognition, and receptive and expressive language across the developmental period of approximately 6–30 months. Families are provided a small kit of basic play materials (rattles, book, bubbles etc.) to support the live (synchronous) session. In practice, this protocol could be used as 1) a second stage screener (e.g. a more specialized screener for infants initially flagged on universal screening tools (Eisenhower et al., 2020; Roberts et al., 2019) to identify infants who would benefit from referral to a specialized evaluation; 2) to support longitudinal behavioral assessment and developmental monitoring; or 3) to monitor treatment progress in parent coaching interventions. Prior work has demonstrated initial reliability and validity of the measures gathered using several metrics: (1) high inter-rater reliability of the AOSI and IT-CC scores, with ICC’s ranging from 0.88 to 0.94; (2) moderate test-retest reliability for AOSI and IT-CC scores, with correlations of 0.46 for AOSI total score and 0.75 for IT-CC total score; (3) moderate correlations between parent ratings of autism symptoms as measured on the Autism Parent Scale for Infants (APSI; Sacrey et al., 2018) and examiner ratings on the AOSI (*r* = .46, *p* = .01). The current study expands on this prior work by qualitatively examining caregivers’ experiences with this specific protocol, with the aim of identifying aspects of the TEDI that are most helpful and most challenging to families in an effort to further refine this specific protocol as well as telehealth-based services for infants and caregivers more broadly. In additional exploratory analyses, we examine whether families from socio-demographic groups most likely to experience digital health inequities rated their experiences differently than groups less likely to experience such inequities.

## Methods

### Participants

Participants included 32 caregivers of infants participating in the TEDI study who enrolled between March 2020 and March 2021. Families were recruited nationwide, and enrolled families lived across 19 different states. All families who completed at least one telehealth session and completed the feedback questionnaire described below during this period were included in the current analysis. Table 1lists the demographic data of the participating families. All participants provided informed consent.

**Table 1:** Sample Demographics

Demographics		Combined Sample (N = 32)
Infant Sex (*n, % male)*		14, 43.8%
Infant Race/Ethnicity (*n*, %)		
	White	28, 87.5%
	Hispanic or Latino	2, 6.3%
	Asian	2, 6.3%
	More than one Race	2, 6.3%
Parent Education (*n*, %)		
	High School/GED/Vocational	2, 6.3%
	Bachelor’s Degree	12, 37.5%
	Master’s Degree	11, 34.4%
	Graduate Degree	7, 21.9%

### Procedure

Families were recruited nationwide for the ongoing study in two sequential cohorts. Recruitment involved postings on family-facing websites (e.g. Institute social media pages, child development organization recruitment pages) and sending the study webpage to early intervention agencies and other providers (e.g. state Part C program coordinators). Many families also self-referred through the study webpage or by contacting the Institute and/or laboratory). Of note, these cohorts represent a convenience sample of highly motivated parents who are not necessarily representative of the national population.

Eligibility criteria for all cohorts included: (1) Infant age between 6 and 12 months at screening; (2) score in the concerns range on any domain of the Infant-Toddler Checklist (Wetherby et al., 2008); (3) English as primary caregiver language; (4) access to a computer or mobile device in the home capable of running the telehealth session; (5) No significant medical (e.g. seizures, head injuries), motor, hearing, or auditory impairments that render the assessment developmentally inappropriate.

Families in Cohort 1 (*n* = 10) participated in a single telehealth assessment visit as part of the initial validation phase from which inter-rate reliability was evaluated. Cohort 2 (*n* = 22) participated in an initial intake and 1-week retest assessment and are currently being followed longitudinally. Cohort 2 infants were assessed using the TEDI session at intake, a one-week retest, and quarterly for 3 additional timepoints. Caregiver feedback for Cohort 2 elicited after the retest session was used in the current analysis. Caregiver feedback for Cohort 1 was elicited following the completion of their single session. Caregiver questionnaires including the APSI (Sacrey et al., 2018) and the Ages and Stages Quesionnaire, 3rd edition (ASQ-3;Squires & Bricker 2006) were completed prior to the telehealth session. The AOSI was scored live by study examiners during the session.

### Feedback Questionnaire

The post-session feedback survey consisted of two parts. First, caregivers completed Likert ratings ranging from 1 (strongly disagree) to 7 (strongly agree) across five dimensions of acceptability on the Telehealth Usability Questionnaire (TUQ), a structured questionnaire adapted to reflect the specific components of the TEDI study (Parmanto et al., 2016). Prior work has reported the mean caregiver ratings for the subdomains of Usefulness, Ease of Use, Effectiveness, Reliability, Satisfaction as well as Overall Mean score, which were each rated significantly more positive than a neutral score using a one-sample Wilcoxon signed rank test comparing the overall mean and subdomain mean scores to a neutral rating of 4 (removed for blinded review). Here we qualitatively analyze caregiver response to four additional open ended questions: (1) were there parts of the TEDI you particularly liked or worked well?; (2) Were there parts of the TEDI that were difficult?; (3) Do you have any suggestions for future versions of this evaluation?; (4) Any additional feedback, comments, or suggestions on any aspect of the assessment? We also quantitatively examined differences between caregivers more or less likely to experience digital health inequities by comparing Overall TUQ scores.

### Coding of Caregiver Responses

Caregiver text responses to all questions were analyzed using an inductive coding process following Braun and Clarke (2006)’s thematic analysis approach. This involved two raters (EL, MT) reviewing all responses to familiarize themselves with the data and generate initial codes. To establish reliability, two raters (EL, MT) independently coded the first five responses and compared their resulting codes. The entire dataset was then coded by a single rater (EL) who also generated initial themes. Codes that were applied to multiple comments from a single caregiver were collapsed into one summary response. For example, a caregiver reporting their infant found the computer distracting under both questions two and four was counted only once when calculating the percentage of caregivers reporting a given theme. Final themes were defined and named via group consensus (MT, SD, EL, FA) and related back to the research literature (all authors).

### Socio-Demographic Characterization

Based on prior literature examining digital health inequities, particularly within the context of COVID-19, we identified four socio-demographic variables in our sample previously linked to lower use or satisfaction with telehealth-delivered healthcare services: income less than $50,000, education less than a bachelor’s degree, rural residence, or Black and/or Latinx self-reported racial/ethnic identity. Rural residence was determined based on the eligible zip codes designated by the Federal Office of Rural Health Policy Data Files (HRSA, 2021). Seven of thirty-two families in our sample belonged to one or more of these groups.

## Results

From our thematic analysis, 14 specific themes emerged within three broad categories (Benefits, Challenges, Suggestions). Responses are summarized below, with some illustrative quotes. Representative excerpts and frequencies for all 14 themes are presented in Table 2. Responses were similar across both cohorts, with only one caregiver in each cohort commenting about the assessment schedule (the component that differed across the cohorts). One parent in Cohort 1 suggested that two visits might “get a better perspective of him” and another parent in Cohort 2 commented that ‘two sessions so close together was a little straining along with our other therapy appointments.”

**Table 2:** Frequency of Each Theme and Representative Excerpts from Caregiver Responses

Response Category	Theme	Percentage of Respondents	Representative Excerpt
Benefits	Provided Materials	53%	I liked getting the box of toys and cue cards. It made me feel prepared going in and had a good understanding of how the appointment would be.
	Clinician Rapport	40%	The researchers were both very professional and insightful, while creating a supportive environment.
	Convenience	37%	I love that it’s Telehealth. We wouldn’t be able to participate in the study otherwise, since we don’t live nearby.
	Session Structure	31%	I really enjoy having two meetings with the researchers, to allow for things to come up that I may have forgotten in a single session or account for unusual behavior/fussiness, etc.
	Representative Infant Behavior	6%	it was very helpful for my child to be home, in his own environment. This made his behavior and reactions genuine and provided an accurate assessment of his abilities.
Challenges	Technology	43%	It was my first go around, but getting the camera angled and moving from area to area with the laptop was a bit challenging.
	Logistics	28%	There is a little bit more leg work for families at the on-set, but nothing that’s not manageable.
	Engaging Child	12%	I think because we were in a familiar setting, my child was less engaged in the activities than he might have been in a different environment.
	Observing Child Challenges	12%	Seeing my baby NOT react to some of the scenarios or asks of the clinicians. That was hard to see as a parent, but it is the reality.
	Length	9%	One caregiver reported that an hour-long session would be enough time, “My son doesn’t like sitting in a high chair for long periods of time so there were a few occasions during the eval where he became very fussy.”
Suggestions	Instructions	21%	The cue cards were helpful, but I think a video example or link to what the test looks like prior to the first session maybe better for us visual learners.
	Preparation	6%	Perhaps it would be a more accurate reflection if we were asked to play with those toys in advance.
	Ongoing Support	6%	Help finding local intervention for those that aren’t able.
	Technology	6%	Maybe do a technology trial run first (if the parent wants). Then I could have figured out camera angles, where I should be, sound, having the assessor’s video off so it wasn’t distracting.

### Response Category One: Benefits

Thirty-one of Thirty-two caregivers reported at least one response falling in the “benefits” category. The 5 themes falling under the Benefits category included: Convenience, Provided Materials, Clinician Rapport, Session Structure, and Representative Infant Behavior. The most commonly reported theme identified by more than half of caregivers related to the study provided materials. Caregivers appreciated the convenience of having all needed materials sent for the session, and described how this facilitated communication with the examiners, who could easily describe the items needed for each activity, saying,

I liked that the clinician could easily tell us what toys to use since the box was provided in advance.

Many caregivers also reported positive rapport with the examiners, despite never having met face-to-face. Finally, many caregivers described the benefits of being able to complete the sessions in their home, without needing to travel, and where they and their infants were most comfortable. For example, one mother noted,

Taking the travel out of the equation is ideal. Especially for parents with children who have sensory issues. This aspect alone is why I would choose telehealth over in-person visits.

### Response Category Two: Challenges

Twenty-seven caregivers reported at least one comment falling into “Challenges.” The 5 themes related to Challenges included: Technology, Logistics, Engaging Child, Observing Child Challenges, and Length. As expected, these reported challenges primarily related to the technological aspects of conducting the session over telehealth. Comments in this theme focused primarily on the need to manage camera angles and on the distraction various devices generated for infants. Under the structural theme, some caregivers described challenges related to the logistics necessitated by the telehealth format. This included taking the lead role in setting up and directing the interactions, and feeling as though communication could be more difficult over video than in person. Whereas two parents described the familiar home environment had been a benefit in gathering a representative sample of infants’ behavior, a handful of parents reported feeling the session had not optimally engaged their child, with one parent noting,

It was somewhat challenging trying to engage my child in the activities in the same fashion as a clinician would (given their unique skillset and my lack of training in this area). I appreciated the suggestions provided over the [device] to help. At times, I worried if I was drawing the same skills out of my child as a trained clinician would be.

Three parents noted it was emotionally difficult to observe their child struggle with some of the tasks presented. One parent shared,

Seeing my baby NOT react to some of the scenarios or asks of the clinician [was challenging]. That was hard to see as a parent, but it is the reality.

### Response Category Three: Suggestions

Thirteen of thirty-two caregivers reported at least one comment falling into “suggestions.” The 4 themes falling under the Suggestions category included: Instructions, Technology, Preparation, and Support. About a quarter of caregivers offered a suggestion falling under the ‘instructions’ theme. Specific suggestions across these themes varied. In terms of Instructions, some caregivers suggested they would have liked a pre-visit video tutorial or a practice session, and one parent thought more explicit instructions for the open-ended play activity would be helpful in eliciting target behaviors. Some families noted a pre-session could have been helpful in identifying locations for filming or the addition of phone stands or the use of Bluetooth audio devices to limit distractions for their infants. Suggestions falling under the Preparation theme included allowing infants to play with session materials ahead of time to reduce their novelty or conducting a “pre-interview.” Suggestions in the Support theme focused on parents’ requests to connect the evaluations with future clinical services (parent coaching or local interventions).

### Influence of Socio-Demographic Variables on Caregiver Satisfaction

We conducted exploratory analyses to examine whether families from socio-demographic groups more likely to experience digital health inequities rated their experience with the TEDI sessions differently than families not from these socio-demographic groups. Although exploratory, mean rank Overall TUQ Scores for caregivers in this group (*Mdn* = 6.36, *n* = 7) were significantly higher than caregivers not in this group (*Mdn* = 5.85, *n* = 25), *U* = 132.00, *p* = .043, *d* = 0.358. Given the exploratory nature of these analyses, this result should be interpreted cautiously.

## Discussion

In this study we gathered feedback from caregivers participating in a study evaluating a telehealth protocol for evaluating infants with early social communication or ASD concerns. Overall feedback was positive, with all but one caregiver reporting at least one positive aspect of the TEDI. The primary benefits identified by caregivers related to the overall ease of access afforded by the telehealth format – not needing to travel, access to research outside of their geographic range, and infants’ behavior representing their usual day-to-day interactions and settings. Caregivers also reported some limitations to this approach. As expected, some caregivers reported the devices used for the session were sometimes distracting, and managing the device and materials increased some parents’ stress associated with the visit. Notably, most of the reported challenges and suggested adaptations reflected individual family/caregiver preferences, rather than universal problems. For example, one parent thought the novelty of the toy kit increased their child’s engagement while another thought the novelty negatively impacted the social interaction. Together, these responses highlight that as with face-to-face evaluations, building rapport, individualization, and working jointly with families are needed as much, if not more, in telehealth format.

Telehealth-based evaluations may be particularly well-suited to evaluations of young infants because evaluation and parent coaching with infants involves such a strong focus on observation of naturalistic parent-child interactions, versus evaluations with older toddlers and children where direct interaction with an examiner via standardized assessment is more routine and developmentally appropriate (Lotzin et al., 2015; Srinivasan et al., 2021; Tesson et al., 2021). While providers have substantially increased the use of telehealth-based observational tools for conducting ASD-related diagnostic assessments with toddlers and older individuals, telehealth-based administration of standardized assessments remains a barrier for the field (Srinivasan et al., 2021; Wagner et al., 2021). Thus, telehealth approaches like we have used here may be most effective when used as a level 2 screener, to increase developmental surveillance, or to develop treatment goals and monitor progress in low-intensity parent coaching interventions.

The current data, and the larger study from which they are drawn, are not without their limitations. First, the sample is a self-selected, high-SES sample which likely does not represent families with the most significant barriers to early telehealth evaluations. Families from lower socioeconomic backgrounds and those living in rural areas are the least likely to have access to in-home broadband, or a tablet or computer (Nadkarni et al., 2020). Care must be taken to ensure translation of services to telehealth does not further exacerbate inequities in healthcare access. More work is needed as a team and as a field to more effectively serve families from diverse cultural and linguistic backgrounds. Our preliminary findings suggest that families in our study with these backgrounds rated the procedures even more acceptable than families from socio-demographic groups with fewer barriers to telehealth-delivered services. This finding likely reflects the fact that families in this study were highly motivated to participate, actively seeking evaluations for their infants, and any barriers they experienced did not prevent them from participating. A second limitation in this work is that examiners in this study were PhD-level psychologists with significant training in developmental evaluations and early ASD identification. More work is needed to evaluate whether such procedures could be carried out by practitioners in community settings. This current analysis represents a first step in developing telehealth-based evaluations that could be integrated into existing community-based programs. Our next steps include more formal procedures, guided by implementation science principles, to adapt and test future iterations of the TEDI in partnership with community-based organizations and stakeholders. Finally, additional work is needed to validate the TEDI against gold-standard, laboratory-based measures.

Better understanding families’ experiences with telehealth-based services can help to identify practices that are desirable and feasible and can help to support sustained used of telehealth-based approaches beyond the COVID-19 pandemic. We found that families appreciated the flexibility, convenience, and natural setting telehealth provides. Families particularly liked receiving a small kit of very basic play materials with written and visual descriptions of the activities to expect during the session. Several families noted that an hourlong session was about the maximum they would like, suggesting that longer comprehensive evaluations may need to be broken into multiple smaller appointments when conducted via telehealth. Families varied in their comfort connecting to the session and managing the video device, with some wanting a practice session and others appreciating the ‘click and go’ approach that minimized time commitments. Overall, feedback from families was overwhelmingly positive. These findings suggest that telehealth-based approaches are a feasible route to increasing families’ access to early screening and evaluations. We believe continued development and validation of the approach described here can help to improve clinical service options and also help to broaden the reach of research beyond the geographic bounds of university-based programs.
